# FOXO1 overexpression and loss of pSerine256-FOXO1 expression predicts clinical outcome in esophageal adenocarcinomas

**DOI:** 10.1038/s41598-018-35459-4

**Published:** 2018-11-26

**Authors:** Katharina Grupp, Faik Güntac Uzunoglu, Nathaniel Melling, Bianca Hofmann, Alexander Tarek El Gammal, Rainer Grotelüschen, Asmus Heumann, Eugen Bellon, Matthias Reeh, Gerrit Wolters-Eisfeld, Tarik Ghabdan, Michael Nentwich, Kai Bachmann, Maximillian Bockhorn, Dean Bogoevski, Jakob Robert Izbicki, Asad Kutup

**Affiliations:** General, Visceral and Thoracic Surgery Department and Clinic, University Medical Center Hamburg-Eppendorf, Hamburg, Germany

## Abstract

The function of Forkhead box O 1 (FOXO1) and pSerine256-FOXO1 immunostaining in esophageal cancer is unclear. To clarify the prognostic role of nuclear FOXO1 and cytoplasmic pSerine256-FOXO1 immunostaining, a tissue microarray containing more than 600 esophageal cancers was analyzed. In non-neoplastic esophageal mucosae, FOXO1 expression was detectable in low and pSerine256-FOXO1 expression in high intensities. Increased FOXO1 and decreased pSerine256-FOXO1 expression were linked to advanced tumor stage and high UICC stage in esophageal adenocarcinomas (EACs) (tumor stage: p = 0.0209 and p < 0.0001; UICC stage: p = 0.0201 and p < 0.0001) and squamous cell carcinomas (ESCCs) (tumor stage: p = 0.0003 and p = 0.0016; UICC stage: p = 0.0026 and p = 0.0326). Additionally, overexpression of FOXO1 and loss of pSerine256-FOXO1 expression predicted shortened survival of patients with EACs (p = 0.0003 and p = 0.0133) but were unrelated to outcome in patients with ESCCs (p = 0.7785 and p = 0.8426). In summary, our study shows that overexpression of nuclear FOXO1 and loss of cytoplasmic pSerine256-FOXO1 expression are associated with poor prognosis in patients with EACs. Thus, evaluation of FOXO1 and pSerine256-FOXO1 protein expression - either alone or in combination with other markers - might be useful for prediction of clinical outcome in patients with EAC.

## Introduction

Esophageal cancer is one of the most aggressive cancers worldwide^1^. Currently, there are limited clinical approaches for the early diagnosis and treatment of esophageal cancer, resulting in a 10% five-year survival rate for patients^1^. Therefore, analysis of novel molecular markers that may help to predict tumor behavior and allow for a personalized therapy in individual esophageal cancer patients are urgently needed. In literature, several biomarkers have been reported in esophageal cancers^2,3^. In EACs, Erb-b2 receptor tyrosine kinase 2 (HER2) has been identified as a relevant prognostic marker which can be targeted by the anti-HER2 monoclonal antibody trastuzumab^4^. Trastuzumab in addition to standard chemotherapy has become standard of care for HER2 positive advanced-stage gastro-esophageal cancers^4,5^. Moreover, a meta-analysis of Creemers *et al*.^2^ showed that several other biomarkers are important in EACs including cyclooxygenase-2, serine/threonine-protein kinase PAK-1, programmed death-ligand 1, MET, insulin like growth factor binding protein 7 and leucine-rich repeat-containing G-protein coupled receptor. Furthermore, prognostic biomarkers have described for the ESCCs. For example, strong evidence supports that epidermal growth factor receptor, Cyclin D1, vascular endothelial growth factor, Survivin, Podoplanin, Fascin, phosphorylated mammalian target of rapamycin, and pyruvate kinase M2 might be significantly linked to patients’ prognosis^3^. This study was performed to get more insights in the prognostic relevance of Forkhead box O 1 (FOXO1) and pSerine256-FOXO1 in esophageal cancers.

The forkhead box O 1 (FOXO1 or FKHR) belongs to the family of Forkhead box O transcription factors, which contain a conserved DNA binding domain and bind a consensus DNA binding sequence TTGTTTAC at target genes^6–8^. FOXO members modulate the expression of genes involved in a broad array of cellular process that include apoptotic cell death, cell cycle control, and DNA damage repair^6,9,10^. FOXO transcriptional activity is negatively regulated by phosphorylation at Serine256 in the PI3K/Akt signaling pathway^11–15^. Phosphorylated forkhead proteins translocate from the nucleus to the cytoplasm where they are inactive^14,16–18^.

In malignancies, the function of FOXO transcription factors is strongly discussed since tumor suppressive^19–25^ as well as oncogenic functions have been reported^26–29^. Earlier IHC studies showed both overexpression and loss of FOXO1 and pSerine256-FOXO1 in malignant cells in comparison to the corresponding benign tissue^30–34^. Additionally, FOXO1 and pSerine256-FOXO1 have been suggested as prognostic markers in malignancies, including breast cancer^30^, bladder^31^, renal cell^32^, prostate cancer^33^, and gastric cancer^34^. However, the prevalence and clinical significance of FOXO1 and pSerine256-FOXO1 expression in esophageal cancer remains elusive. To gain more insights in the potential clinical utility of FOXO1 and pSerine256-FOXO1 protein analysis in esophageal cancer, we used our tissue microarray of more than 600 esophageal cancer specimens with clinical follow-up data.

Our study shows that FOXO1 overexpression and loss of pSerine256-FOXO1 expression are associated with poor prognosis in esophageal adenocarcinomas. Thus, it can be speculated that the evaluation of FOXO1 and pSerine256-FOXO1 in tumor biopsies might be of clinical relevance in patients with EACs.

## Results

### Technical issues

A total of 78.2% and 76.9% of EACs and 81.9% and 79.9% of ESCCs were interpretable for analysis of nuclear FOXO1 and cytoplasmic pSerine256-FOXO1 immunostaining. Non-informative cases were caused by unequivocal malignant tissue or missing tissue spot.

### FOXO1 and pSerine256-FOXO1 expression in benign and neoplastic esophageal tissue samples

**Figure 1:**
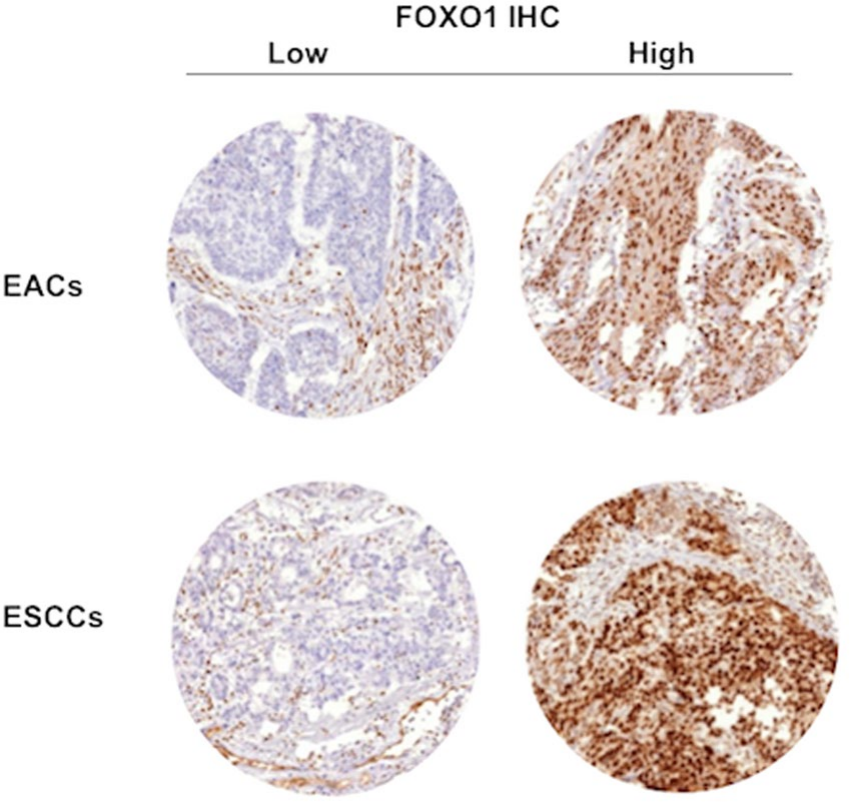
Representative pictures of low and high FOXO1 IHC in esophageal cancers.

FOXO1 expression was predominantly localized in the nucleus of the cells. FOXO1 immunostaining was detectable - if present - in weak intensities in stratum basal cells of the non-neoplastic esophageal mucosa. Cancer cells showed increased levels of FOXO1 expression compared to benign esophageal cells. High FOXO1 expression was found in 40.2% of EACs and 45.2% of ESCCs. Representative images of FOXO1 immunostaining in esophageal cancers are given in Fig. 1.

**Figure 2:**
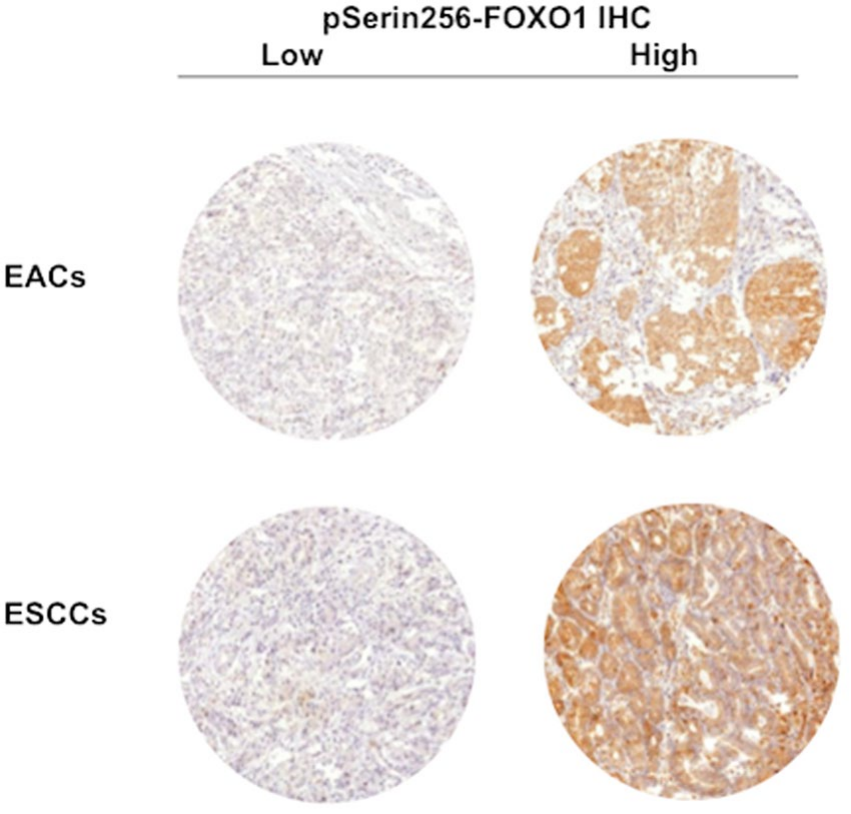
Representative pictures of low and high pSerine256-FOXO1 IHC in esophageal cancers.

Expression of pSerine256-FOXO1 was predominantly localized in the cytoplasm of the cells and was found in decreased intensities in malignant compared to benign esophageal epithelium. Low pSerine256-FOXO1 immunostaining was found in 59.8% of EAC and 37.4% of ESCC samples. Representative images of pSerine256-FOXO1 expression in malignant esophageal tissue are shown in Fig. 2.

### Overexpression of FOXO1 and loss of pSerine256-FOXO1 expression are associated with unfavorable tumor phenotype in esophageal cancers

The associations of FOXO1 and pSerine256-FOXO1 expression with tumor phenotype are shown in Tables 1 and 2. Increased FOXO1 and decreased pSerine256-FOXO1 expression were significantly associated with advanced tumor stage and high UICC stage in both EACs (tumor stage: p = 0.0209 and p < 0.0001; UICC stage: p = 0.0201 and p < 0.0001) and ESCCs (tumor stage: p = 0.0003 and p = 0.0016; UICC stage: p = 0.0026 and p = 0.0326). Additionally, overexpression of FOXO1 and loss of pSerine256-FOXO1 expression were linked to presence of lymph node metastases in the subset of ESCCs (p = 0.0028 and p = 0.0119).

**Table 1:** Associations of FOXO1 and pSerine256-FOXO1 IHC results and clinic- pathological features of EACs.

	FOXO1				pSerin256-FOXO1			
	Analyzable, n	Low, %	High, %	P value	Analyzable, n	Low, %	High, %	P value
All cancers	281	59.79	40.21		276	59.78	40.21	
Age group								
<65 years	94	62.77	37.23	0.4692	91	62.64	37.36	0.4966
>65 years	187	58.29	41.71		185	58.38	41.62	
Sex								
male	239	59	41	0.5167	232	59.48	40.52	0.6832
female	42	64.29	35.71		43	62.79	37.21	
Tumor stage								
pT1	55	74.55	25.45	0.0209	61	32.79	67.21	<0,0001
pT2	32	59.38	40.63		29	58.62	41.38	
pT3	175	57.71	42.29		168	68.45	31.55	
pT4	17	35.29	64.71		16	75	25	
UICC stage								
I	55	74.55	25.45	0.0201	59	33.9	66.1	<0,0001
II	39	61.54	38.46		36	69.44	30.56	
III	162	52.47	47.53		159	67.3	32.7	
IV	23	69.57	30.43		20	65	35	
Tumor grading								
G1	16	93.75	6.25	0.0071	16	31.25	68.75	0.1046
G2	100	54	46		105	63.81	36.19	
G3	157	59.87	40.13		146	59.59	40.41	
G4	5	80	20		6	66.67	33.33	
Resektion margin								
R0	207	61.35	38.65	0.1464	204	59.8	40.2	0.6266
R1	67	55.22	44.78		65	61.54	38.46	
R2	3	100	0		3	33.33	66.67	
Lymph node metastasis								
N0	84	69.05	30.95	0.1327	85	44.71	55.29	0.0063
N1	47	59.57	40.43		47	70.21	29.79	
N2	65	58.46	41.54		64	64.06	35.94	
N3	82	51.22	48.78		79	67.09	32.91	
Distant metastasis								
M0	258	58.91	41.09	0.3106	256	59.38	40.63	0.6186
M1	23	69.57	30.43		20	65	35

**Table 2:** Associations of FOXO1 and pSerine256-FOXO1 IHC results and clinico-pathological features of ESCCs.

	FOXO1					pSerin256-FOXO1			
	Analyzable, n	Low, %	High, %	P value		Analyzable, n	Low, %	High, %	P value
All cancers	208	54.81	45.19			203	37.44	62.56	
Age group									
<65 years	76	53.95	46.05	0.8043		74	40.54	59.46	0.5161
>65 years	131	55.73	44.27			128	35.94	64.06	
Sex									
male	152	51.97	48.03	0.134	147		40.82	59.18	0.1212
female	55	63.64	36.36		55		29.09	70.91	
Tumor stage									
pT1	35	85.71	14.29	0.0003	36		11.11	88.89	0.0016
pT2	40	52.5	47.5		37		45.95	54.05	
pT3	121	46.28	53.72		117		41.88	58.12	
pT4	12	58.33	41.67		13		46.15	53.85	
UICC stage									
I	49	73.47	26.53	0.0026	49		24.49	75.51	0.0326
II	54	61.11	38.89		51		31.37	68.63	
III	95	42.11	57.89		93		46.24	53.76	
IV	9	55.56	44.44		9		55.56	44.44	
Tumor grading									
G1	3	33.33	66.67	0.1667	2		50	50	0.1266
G2	132	59.85	40.15		127		32.28	67.72	
G3	72	47.22	52.78		73		46.58	53.42	
G4	0	0	0		0		0	0	
Resektion margin									
R0	153	58.82	41.18	0.1115	144		36.11	63.89	0.7251
R1	46	41.3	58.7		48		41.67	58.33	
R2	7	57.14	42.86		9		44.44	55.56	
Lymph node metastasis									
N0	92	69.57	30.43	0.0028	91		26.37	73.63	0.0119
N1	49	42.86	57.14		43		41.86	58.14	
N2	40	42.5	57.5		40		55	45	
N3	25	48	52		27		44.44	55.56	
Distant metastasis									
M0	199	54.77	45.23	0.6645	194		37.11	62.89	0.4677
M1	8	62.5	37.5		8		50	50	

### High FOXO1 and low pSerine256-FOXO1 expression predict shortened survival in EACs

**Figure 3:**
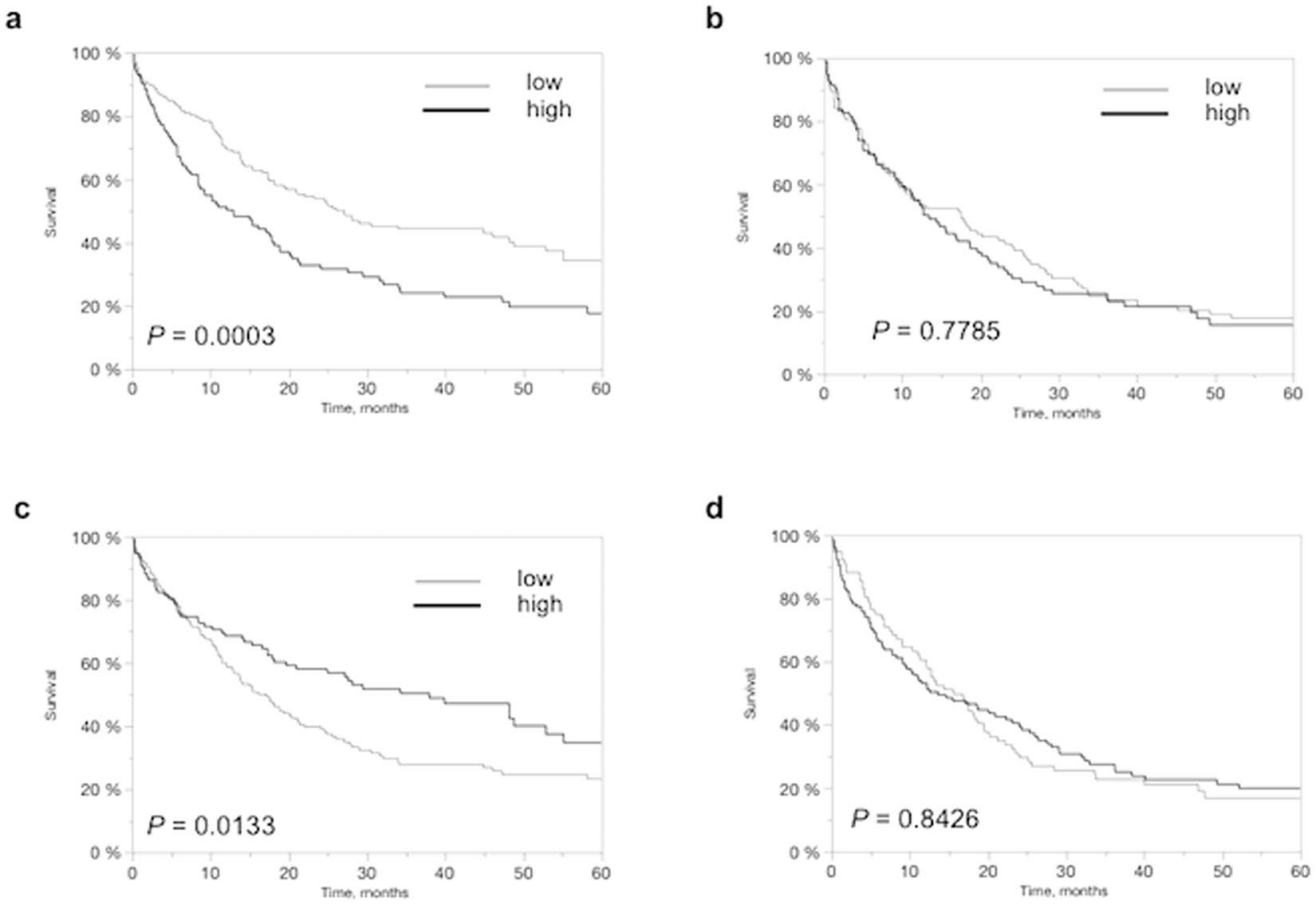
Clinical impact of FOXO1 and pSerine256-FOXO1 IHC. Relationship of FOXO1 immunostaining intensity with overall survival in EACs (n = 281; *P* = 0.0003; (**a**) and ESCCs (n = 207; *P* = 0.7785; (**b**). Association of pSerine256-FOXO1 immunostaining intensity with overall survival in EACs (n = 276; *P* = 0.0133; (**c**) and ESCCs (n = 202; *P* = 0.8426; (**d**).

Kaplan Meyer curves demonstrated that high FOXO1 and low pSerine256-FOXO1 expressions were associated with shortened survival of patients with EACs (p = 0.0003 and p = 0.0133) but were unrelated to clinical outcome in patients with ESCCs (p = 0.7785 and p = 0.8426), as demonstrated in Fig. 3.

**Figure 4:**
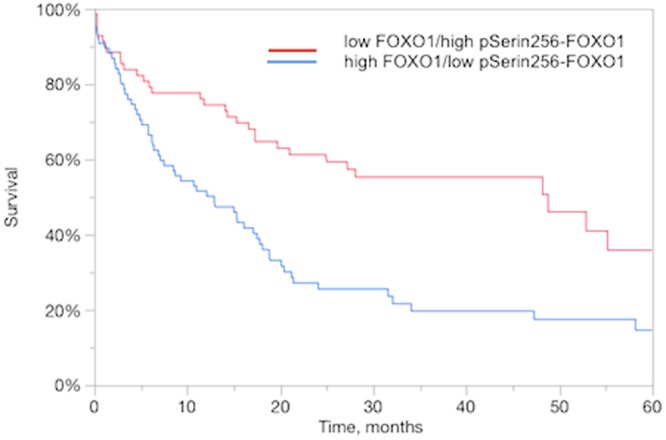
Clinical impact of combined FOXO1 and pSerine256-FOXO1 IHC in the subset of EACs. Relationship FOXO1 low/ pSerine256-FOXO1 high and FOXO1 high/ pSerine256-FOXO1 low immunostaining intensity with overall survival in EACs (n = 92; P = 0.0002).

Additionally, we analyzed the clinical impact of the combination of both staining and demonstrated that the combination of immunostainings was significantly associated with clinical outcome of patients (p = 0.0002; Fig. 4). The group of patients with high FOXO1 and low pSerine256-FOXO1 expressions was significantly linked to worse outcome in EACs as shown in Fig. 4. Overall, the combined IHC staining (FOXO1/pSerine256-FOXO1 expression) predicted more effective the 1-year (p = 0.0004), 2-year (p = 0.0001) and 3-year (p = 0.0001) survival than the analysis of a single IHC staining (FOXO1: 1-year: p = 0.003; 2-year: p = 0.0096; 3-year: p = 0.0003 and pSerine256-FOXO1: 1-year: p = 0.0254; 2-year: p = 0.002; 3-year: p = 0.0063) staining.

### Multivariate analysis including FOXO1 expression, pSerine256-FOXO1 expression, and the combination of FOXO1/pSerine256-FOXO1 expression

Multivariate analysis including tumor stage, UICC stage and FOXO1 IHC demonstrated that tumor stage, UICC stage and FOXO1 IHC were independent prognostic markers (p = 0.0173, p < 0.0001, and p = 0.002). Moreover, analysis including tumor stage, UICC stage and pSerine256-FOXO1 showed independent significant results for tumor stage and UICC stage but not for pSerine256-FOXO1 expression status (p = 0.0186, p < 0.0001 and p = 0.5394). Furthermore, we performed multivariate analysis including tumor and UICC stage and the group FOXO1/ pSerine256-FOXO1 IHC. In this analysis, all of these factors showed independent prognostic significance (p = 0.0245, p < 0.0001, and p = 0.0176).

## Discussion

Our study shows that overexpression of nuclear FOXO1 and loss of cytoplasmic pSerine256-FOXO1 expression are associated with poor prognosis in patients with EACs. Thus, analysis of FOXO1 and pSerine256-FOXO1 expression - either alone or in combination with other markers - might be useful for prediction of clinical outcome in these patients.

Here, we evaluated FOXO1 and pSerine256-FOXO1 expressions in malignant and benign esophageal tissue samples on TMAs using immunohistochemistry. Earlier, it has been hypothesized that analysis of correlations between molecular markers and survival is limited due to the fact of tumour heterogeneity^35^ and that analysis of multiple cores per tumor specimen would enhance the representativity of TMA studies^36^. This suggestion is based on the assumption, that concordance of large section findings with tissue microarray data is better if 3–4 cores are taken per cancer sample is taken than just only one core per tumor sample. However, these ideas are based on the assumption that there exist a significant heterogeneity within the tissue represented by a standard 3 × 4 cm paraffin block, and that tumor heterogenity is adequat estimated by the analysis of large section. In our view, these hypotheses are open to debate. Previously, it has been shown that the TMA format is generally superior over large section studies to analyse relationships between molecular markers and clinical outcome^37^. In detail, TMA and large section findings of p53, PR, and ER in breast cancer were compared and in summary the results showed that overinterpretation of focal p53 positivity in large sections obscured the established prognostic impact of p53, which was, however, significantly estimated in the TMA analysis^37^. Further analyses demonstrated comparable significant relationships between Ki67 or p53 expression and aggressive prostate tumor features if three tissue cores were separately studied or if a combined result was done from the three cores^38^. In our opinion, these studies demonstrated that usage of multiple cores does not necessarily increase the ability to identify relationship between biomarkers and clinico-patholgical parametes. Moreover, these results underline the robustness of IHC TMA studies for analysis of correlations of molecular markers with clinico-pathological features of cancer specimens.

Here, we analyzed FOXO1 and pSerine256-FOXO1 expression in esophageal cancers. In our study, FOXO1 expression was found in increased intensities and pSerine256-FOXO1 expression in decreased intensities in malignant than in benign esophageal tissue. High FOXO1 and low pSerine256-FOXO1 staining occurred in 40.2% and 59.8% of EACs and 45.2% and 37.4% of ESCCs. Our observation of aberrant FOXO1 expression in cancerous relative to non-cancerous esophageal tissue is consistent with earlier studies on FOXO1 expression in diverse other cancer types, such as bladder^31^, renal cell^32^, breast^30^, and prostate cancer^33^. However, inconsistently, these immunohistochemically studies suggested either an increased or a decrease of FOXO1 expression in malignant relative to corresponding benign tissue^31–33^. Possible explanations for differing expression status of FOXO1 in different tumor types include variable interactions with critical pathways depending on the spectrum of tissue type specific gene activation.

Our data demonstrate that FOXO1 overexpression and loss of pSerine256-FOXO1 expression are linked to a subset of esophageal cancers with aggressive tumor features. Of importance, the prognostic impact of FOXO1 and pSerine256-FOXO1 were limited to the histological subset of EACs, while the markers were unrelated to clinical outcome in ESCCs. Moreover, our data suggest that even the measurement of both IHC markers FOXO1 and pSerine256-FOXO1 might be in combination of clinical relevance. This observation underlines an important role of FOXO1 and its phosphorylated form in EACs which may also be due to above mentioned tissue-specific gene activation.

The majority of EACs are believed to develop from the precursor lesion (metaplastic glandular esophageal epithelium/Barrett’s oesophagus) evolving through a sequence from low grade, to high grade dysplasia and eventually to carcinoma^39,40^. However, the driving factors for progression are still incompletely understood^39,40^. Although several genetic and cellular changes have been described, none of these as yet have proven utility^41,42^. However, hallmarks of metaplastic Barrett’s oesophagus are increased proliferation and decreased apoptosis and it is believed that these changes are important in malignant progression by increasing the vulnerability to, and perpetuation of mutations^39,40^. FOXO1 gene is involved in several biological functions of cancer cells such as cell proliferation, apoptosis, cell differentiation, and angiogenesis^43^. These cellular processes are known to be closely linked to tumorigenesis, and FOXOs play central roles in the regulation of cell proliferation and cycle by regulating several genes such as p53, p27Ki p1, cyclin B, cyclin D1/D2, and cyclin G2^9,43,44^.

Additionally, other signaling pathways linked to cell proliferation, survival, migration, invasion and angiogenesis are known to play important roles in esophageal tumorigenesis. For example, PI3K-Akt signaling pathway, which also regulates the forkhead family transcription factors^14,45^, is a key pathway involved in esophageal tumorigenesis carcinogenesis^46^. Dysregulation of PI3K-Akt signaling pathway has been associated with increase cancer cell growth, proliferation, migration and invasion in esophageal cancers in literature^47–49^. Previously, several studies analysing the genomic landscape described an association between frequent disruption of pathways linked to important cellular signaling pathways including proliferation, survival, invasion, apoptosis, and migration. For example, studies found that dysregulation of MAPK signaling, PI3K-Akt signaling, and wnt signaling were strongly involved in esophageal tumorigenesis^50–52^. It can be speculated that FOXO1 and pSerine256-FOXO1 as well as its regulating signaling pathways might be important roles during esophageal carcinogenesis.

In earlier studies on FOXO1 expression in other cancer types, high FOXO1 expression was suggested to be a prognostic marker for improved clinical outcome in breast^30^, lung^53^ and bladder^31^ cancers. However, our data suggests that FOXO1 might play an oncogenic role in esophageal cancers. Functional data on FOXO proteins in different cancer types are conflicting. Some authors suggested FOXO factors as tumor suppressors in some cancer types^54^, while others reported on oncogenic roles of FOXO proteins^55,56^. In detail, FOXO factors can positively regulate cell survival and resistance to chemotherapy, complicating its putative therapeutic potential^54^. For example, FOXO3a is a positive regulator of androgen receptor expression and prostate cancer cell proliferation^55^. In addition, loss of functional FOXO3a in human ovarian cancer cell lines limited the sensitivity of ovarian cancer cells to chemotherapy, suggesting that FOXO proteins may be responsible for altered treatment outcomes in the presence of combined therapeutic approaches^56^. Taken together, greater understanding of the function and regulation of FOXO proteins are still needed to fully understand the role of FOXO proteins during carcinogenesis. Further studies analysing the functional roles of FOXO proteins are necessary to fully elucidate the role of FOXO proteins in esophageal cancer development and progression.

Previously, studies analysing whole-genome and whole-exome sequences from tumor specimens identified mutations that are enriched in tumor samples compared to germline cells. It is widely accepted that these mutations are the main drivers of tumor progression^57^. It is believed that cancers result from a combination of perturbed genes acting in molecular networks that correspond to hallmark processes such as cell proliferation and apoptosis^58^. In detail, mutations in signaling proteins may over-enrich key signaling pathways or inhibit the function of tumor suppressor proteins, resulting in uncontrolled cell growth, tumor development and progression^59^. In this study, we identified FOXO1, known to be involved in key signaling pathways, as an additional deregulated marker linked to prognosis of patients with esophageal cancers.

In summary, our study shows that increased FOXO1 immunostaining is marginally linked to aggressive tumor features in esophageal cancer but is unrelated to survival of patients. Therefore, our study excludes FOXO1 as prognostic EAC biomarker.

## Methods

### Esophageal cancer TMA

A TMA was constructed from cancer tissues after radical esophagectomies from 359 esophageal adenocarcinoma patients and 254 esophageal squamous cell carcinoma patients treated at the Department of General, Visceral and Thoracic Surgery at the University Medical Center Hamburg-Eppendorf. Follow-up was available of 359 esophageal adenocarcinoma patients and 254 esophageal squamous cell carcinoma patients. Median follow-up was 17.3 (range: 0 to 208) and 12.2 months (range: 0 to 191 months) in esophageal adenocarcinoma and esophageal squamous cell carcinoma patients. TMAs were manufactured as described^60^.The study was approved by the Ethics commission Hamburg and conducted in accordance with the Declaration of Helsinki. Informed consent has not been collected specifically for the patient samples included in this study. Usage of routinely archived formalin fixed leftover patient tissue samples for research purposes by the attending physician is approved by local laws and does not require written consent (HmbKHG, §12,1).

### Immunochemistry

Primary antibody for FOXO1 (rabbit, Cell signaling) was applied at a dilution of 1:150 and for pSerine256-FOXO1 (rabbit, Abcam) at a dilution of 1:450 according to the manufacturer´s directions. FOXO1 and pSerine256-FOXO1 staining were analyzed in immunohistochemisty. Visualization of the primary antibody was performed with the EnVision Kit (Dako, Glostrup, Denmark). FOXO1 and pSerine256-FOXO1 staining were homogenous in the analyzed tumor samples and staining intensity of all cases was thus semiquantitatively assessed in the following two categories: low and high immunostaining. All methods were carried out in accordance with relevant guidelines and regulations.

### Statistical analysis

Statistical calculations were performed using JPM 9 software (SAS Institute Inc., NC, USA). To analyze association between IHC results and clinico-pathological features contingency tables were used and tested with the chi-square method. Kaplan-Meier curves were generated for survival analysis. Log-rank test was applied to check significant survival differences between groups. Cox proportional hazards regression analysis was performed to test for independence and significance between pathological, molecular, and clinical variables.

### Ethical approval and informed consent

The study was approved by the Ethics commission Hamburg and conducted in accordance with the Declaration of Helsinki. Informed consent has not been collected specifically for the patient samples included in this study. Usage of routinely archived formalin fixed leftover patient tissue samples for research purposes by the attending physician is approved by local laws and does not require written consent (HmbKHG, §12,1).

## Data Availability

All data generated or analysed during this study are included in this published article.
